# Access and cost of services for autistic children and adults in Italy: a carers’ perspective

**DOI:** 10.3389/fpsyt.2024.1299473

**Published:** 2024-03-12

**Authors:** Martina Micai, Francesca Fulceri, Tommaso Salvitti, Giovanna Romano, Maria Luisa Scattoni

**Affiliations:** ^1^ Research Coordination and Support Service, Istituto Superiore di Sanità, Rome, Italy; ^2^ Directorate General of Health Prevention, Ministry of Health, Rome, Italy

**Keywords:** autism spectrum disorder, use of services, financial cost, carers, Italy

## Abstract

**Background:**

Limited information exists on autistic service access and costs in Italy.

**Objectives:**

This study aims to investigate access to educational, healthcare, social, and related services for autistic individuals in Italy as part of the Autism Spectrum Disorder in the European Union (ASDEU) project.

**Methods:**

Italian carers of autistic individuals completed an online survey regarding services and costs in the 6 months before completion.

**Results:**

Three hundred and three carers of autistic people participated in the survey. The majority of those receiving care were children, males, and lived at home with their parents. Autistic adults were often students (17%) or unemployed but willing to work (17%). Employed carers (49%) worked on average 32.23 ± 9.27 hours per week. A significant portion (82%) took work or school absences to care for autistic individuals, averaging 15.56 ± 14.70 days. On average, carers spent 58.84 ± 48.36 hours per week on caregiving duties. Fifty-five of the autistic individuals received some form of support, 5% utilized residential care, and 6% were hospitalized. Thirty-four percent received outpatient hospital care, and 20% underwent some form of autism-related psychopharmacological therapy. School support was primarily provided by support teachers (18.16 ± 7.02 hours/week). Educational psychologists (80.73%), psychomotor therapists/physiotherapists (53.85%), and speech therapists (50.91%) were frequently paid by carers who paid more per hour. Autistic children received support from educators (73.96 hours/week), group therapy (32.36 hours/week), and speech therapists (31.19 hours/week). Psychologists (76.00%) and counseling/individual therapists (89.13%) were often paid by carers. Carers reported high costs for psychiatrists and psychologists, with frequent use of psychiatric services (8 ± 8 times in 6 months).

**Conclusions:**

Carers’ perspectives on the access and costs of services for autistic individuals in Italy can provide insights into areas for improvement in the delivery of autism services.

## 1 Introduction

Autism Spectrum Disorder (ASD) is a neurodevelopmental condition characterized by difficulties in communication and social interaction (DSM-5, 1). It is estimated that globally, approximately one in 100 children is diagnosed with ASD (2). The prevalence of ASD has increased over the past few decades, now concerning over 70 million individuals worldwide. In Italy, it is estimated that 1 in 77 children (ages 7-9 years) has ASD, with a higher prevalence in males, who are 4.4 times more likely than females (3). The rising number of ASD diagnoses, coupled with the lifetime nature of the condition requiring ongoing educational, health, living, and social support, underscores the significant costs faced by individuals, families, and the healthcare systems. The cost of caring for autistic individuals can be substantially varying based on factors such as the severity of the condition, co-occurring conditions, presence of intellectual disability, age, and type of required services. Rogge and Janssen (4) reviewed approximately 50 studies exploring costs associated with medical and healthcare services, therapies, education, production loss for autistic adults, informal care, lost productivity for family/carers, accommodation, respite care, and out-of-pocket expenses. Most studies were conducted in the US and UK, revealing costs of $2.4 million and $2.2 million for an autistic individual with an intellectual disability in the US and the UK, and $1.4 million for both scenarios without an intellectual disability (4). A study by Vohra and colleagues (2013), interviewing 2,936 carers of autistic children aged 3–17 years, observed that carers of autistic children faced challenges in utilizing services, lacked a reliable care source, experienced insufficient insurance benefits, encountered a lack of collaborative decision-making and care synchronization, and reported adverse effects on the family, in contrast to carers of children with other developmental disabilities or mental health issues (5). Regarding medical care, autistic individuals often require specialized services such as speech therapy, occupational therapy, and psychological counseling. In addition to direct costs, autistic individuals and their families also incur indirect costs, including lost wages and reduced productivity. For instance, parents of autistic children may need to take time off work to care for their children, impacting their earnings and economic stability. These challenges can affect the mental health and quality of life of carers of autistic individuals (6). Autistic adults may also encounter challenges in finding and maintaining employment, further impacting their financial stability.

Italy has a national health service (Servizio Sanitario Nazionale – SSN), with public healthcare primarily funded through national and regional taxes, supplemented by patient co-payments (7). While recognizing that ASD imposes a significant public health cost in Italy, there is limited information available regarding the burden and services utilized for this condition. Despite the high costs associated with ASD, numerous services are accessible to support autistic individuals and their families. These services encompass government-funded programs, private organizations, and community-based support groups. As shown in the survey-based study conducted by Mendez and colleagues (8), a majority of ASD interventions in Italy are publicly funded (42.62%). Standard offerings include educational programs, job training, and housing support.

This survey-based study aims to understand the costs and services utilized for ASD in Italy, as well as the caregiving responsibilities, by exploring the experiences of carers of autistic children and adults. This method was selected based on its ability to efficiently gather a diverse range of perspectives from carers in a cost-effective and feasible manner through remote administration. On the one hand, it is important to consider that administering a survey to carers allows for insights into their perspectives on this topic rather than those of autistic individuals. On the other hand, it proves valuable in collecting experiences from carers of individuals who may have more difficulty in expressing themselves.

## 2 Methods

### 2.1 Survey development

Italian carers of autistic individuals completed an online survey of the utilization of services dedicated to ASD and associated costs in the 6 months leading up to the survey completion. The survey, originally developed in English for prior studies conducted in Scotland (9), was translated into Italian and adapted to fit the Italian education, healthcare, and social care systems. A new section focusing on medication use was also added. The Italian version of the survey can be found in **Supplementary Data 1**.

### 2.2 Survey description

The survey is designed to gather information on access to education, healthcare, and social services, and it is intended to be anonymously completed by carers of autistic individuals of all ages. The estimated time to complete the survey is approximately 20-30 minutes. The survey covers the following topics:

1. Demographic information about the autistic individual, including age, gender, nationality, country of residence, current region residence, subtype of ASD, any other conditions diagnosed by a doctor, intelligence quotient, and the score achieved in the most recent intelligence test, if available.
2. Living accommodation details of the autistic individual, specifying their current living situation (private household with parents or relatives, private household with friends/flatmates, private household with a partner, private household alone, etc.).
3. Employment status of autistic individuals aged 16 and over who have left school. If employed, the survey inquiries about the number of hours worked per week in paid or unpaid employment and whether there have been any absences in the last 6 months due to ASD.
4. Educational services utilized by autistic individuals who have not left education and have accessed educational services. The survey explores the educational services attended in the last 6 months, the professionals at a school or university seen by the individual in the last 6 months, the number of hours per week, whether the carer or individual paid for any of these services directly (using personal funds or supported by a benefit or allowance) and an indication of the cost in euros if known.
5. Information on the type of tuition/tutorial support, residential respite care (temporary institutional care providing relief for the usual carers of autistic person), and outpatient hospital care (visits to a hospital, clinic, or associated facility for diagnosis or treatment) received by the autistic person in the 6 months, along with the number of days spent in residential respite care and the number of times services were used in the 6 months leading up to the survey completing the survey completion. If the carer or individual paid for the service directly, the survey also inquired about the cost per day in euros. Additionally, the survey asked if the autistic person received any support in the 6 months, including the number of visits, the average length of visits, whether the support was paid directly by the carer or individual, and the associated cost.
6. Information on medication related to ASD symptoms and other conditions associated with ASD in the 6 months, including the name of the drug, dosage, dose frequency, and the duration for which the person took this drug in the 6 months before completing the survey (reported in the number of weeks).
7. Employment status of the carer. The survey inquired about the number of hours per week the carer spent caring for the autistic person and whether the carer used any health or social care services for himself/herself over the last 6 months due to caring for the autistic person. This includes the number of times the services were used, the duration in hours if the service was paid for directly by the carer, and the associated cost per visit.

### 2.3 Sample recruitment and survey distribution

The survey was launched using the LimeSurvey tool (http://www.limesurvey.org) in February 2017. Survey notices and invitations to participate were sent to ASD organizations (both national and local level) and service provider organizations (both public and private, including residential facilities, job training, and education programs). These organizations were also encouraged to share the survey links through communication channels such as e-newsletters, websites, and social media accounts. Additionally, researchers disseminated the survey through their professional networks and social media platforms. This recruitment approach was chosen due to the limited resources available for the ASDEU study. Data for this analysis were based on responses obtained until February 2018. Ethical approval for survey distribution in Italy was obtained from the National Institute of Health [Istituto Superiore di Sanità] (Protocol number PRE 172/16). All procedures in studies involving human participants adhere to the ethical standards of the institutional and/or national research committee, as well as with the World Medical Association Declaration of Helsinki and its subsequent amendments or comparable ethical standards. Prior to starting the survey, respondents were required to read the provided information and sign electronic informed consent. No personal identifying information was collected. For analysis, data were handled in aggregated form; participants did not receive feedback, and individual respondent results were not reported. The survey’s background information section gathered a few demographic characteristics to classify the respondents for analysis purposes, including age, gender, nationality, country of residence, and employment status.

### 2.4 Analysis methods

Analyses were conducted for surveys that participants had completed. Aggregated descriptive statistics were calculated for all questions, including the mean and standard deviation (SD) for continuous data and proportions for categorical data. The distribution of responses (frequency and percent) from the respondent group is presented. Analyses for children and adults (≥18 years old) were performed and provided separately for all the questions. To highlight potential differences between the means of the children’s group and the adults’ group, Student’s t-tests were conducted with a sample size of at least ten respondents per group. With a smaller number of observations, the Student’s t-test was performed under the condition that the two groups had similar variance and the data met the normality condition. When the data was not normally distributed, the Wilcoxon Mann-Whitney test was used. The Student’s t-test and Wilcoxon test were not conducted whenever a statistical power issue was identified (e.g., only one unit per group).

## 3 Results

A total of 303 carers of autistic individuals participated in the survey. The majority of those under care were children under 18 years (82%), with an average age of 11.61 ± 8.24 years (range: 1-49). Most were male (82%) and Italian nationals (95%). The distribution of participants’ origins across Italian regions was as follows: Lazio (15%), Emilia-Romagna (12%), Lombardy (10%), Campania (9%), Sicily, Tuscany, Piedmont, Veneto (7% each), and Puglia (6%). Nine percent of the autistic individuals were diagnosed with Asperger Syndrome, and the rest with ASD and Generalized Developmental Disorder - not otherwise specified. The majority of the co-occurring conditions reported were intellectual disabilities (23%), sleep disturbances (22%), attention deficit hyperactivity disorder (ADHD, 20%), anxiety (19%), and gastrointestinal disorders (19%). Of the 81 (27%) carers who responded to this question, 73% of the autistic individuals scored between 71 and 152 on the intelligent quotient tests, while the rest scored below 70. No one specified the intelligence quotient test used for the assessment. The majority of autistic individuals lived at home with their parents (97%). Most autistic adults were full-time students and did not engage in any work activity (17%), were unemployed but willing to work (17%), or were unemployed and not willing to work (10%). The educational services most frequently accessed by autistic people were preschools, elementary schools, and junior high schools (69%), high school/technical schools (12%), and support classrooms inside the schools (7%).

Almost half of the carers were employed (49%), working an average of 32.23 ± 9.27 hours per week [range: 3-50]. 82% of the carers took some absences from work or their place of study in the last 6 months to take care of an autistic person, for an average of 15.56 ± 14.70 days [1-120]. On average, carers reported spending 58.84 ± 48.36 hours per week [range: 2-168] caring for the autistic person. The percentage of carers who claimed providing care for the autistic individual for 2 to 10 hours was 14.02% (n=37), whereas those caring for more than 151 hours was 5.68% (n=15).

### 3.1 Service access frequency

**Table 1** shows the results concerning the access and use, in the last 6 months of the investigated services.

**Table 1:** Service access frequency over the last six months.

	Children (n=247)		Adults (n=56)		Overall (n=303)	
	N (%)*	Mean (SD)	N (%)*	Mean (SD)	N (%)*	Mean (SD)
Educational services (hours per week)						
Educational psychologist	102 (41.30)	5.38 (5.69)	6 (10.71)	8.60 (10.97)	108 (35.64)	5.56 (6.06)
Social worker	20 (8.10)	7.40 (4.22)	1 (1.79)	16.00 (0.00)	21 (6.93)	7.81 (4.52)
Special classroom assistant	82 (33.20)	9.60 (6.49)	7 (12.50)	9.86 (3.98)	89 (29.37)	9.61 (6.32)
Support teacher	200 (80.97)	18.16 (7.02)	9 (16.07)	16.78 (8.45)	209 (68.98)	18.10 (7.07)
Speech therapist	52 (21.05)	2.13 (1.39)	1 (1.79)	1.00 (0.00)	53 (17.49)	2.11 (1.38)
Occupational therapist	12 (4.86)	4.67 (3.34)	–	–	12 (3.96)	4.67 (3.34)
Psychomotor therapist/physiotherapist	41 (16.60)	2.09 (1.60)	1 (1.79)	2.00 (0.00)	42 (13.86)	2.09 (1.58)
Basic assistant	41 (16.60)	11.40 (7.86)	2 (3.57)	5.00 (1.41)	43 (14.19)	11.10 (7.79)
Other	75 (30.36)	8.26 (8.22)	15 (26.79)	4.62 (5.43)	90 (29.70)	7.65 (7.91)
Individual lessons at home	62 (25.10)	5.06 (4.00)	8 (14.29)	5.63 (6.05)	70 (23.10)	5.12 (4.23)
Individual lessons at school	16 (6.48)	5.34 (5.35)	4 (7.14)	5.50 (1.00)	20 (6.60)	5.37 (4.77)
Small group lessons at school	13 (5.26)	3.54 (2.18)	2 (3.57)	5.00 (1.41)	15 (4.95)	3.73 (2.12)
Other	43 (17.41)	6.45 (8.15)	6 (10.71)	15.50 (13.33)	49 (16.17)	7.56 (9.25)
Services for carers (N of times)						
Additional visit with family doctor	10 (4.05)	3.10 (1.79)	3 (5.36)	9.33 (12.70)	13 (4.29)	4.54 (6.06)
Family planning	5 (2.02)	2.28 (0.84)	–	–	5 (1.65)	2.28 (0.84)
Social Services	12 (4.86)	4.25 (6.78)	4 (7.14)	1.50 (0.58)	16 (5.28)	3.56 (5.94)
Psychiatric Services	12 (4.86)	8.42 (8.27)	2 (3.57)	2.00 (1.41)	14 (4.62)	7.50 (7.97)
Marriage counselor	4 (1.62)	6.75 (4.57)	–	–	4 (1.32)	6.75 (4.57)
Counseling	12 (4.86)	4.71 (3.06)	2 (3.57)	6.50 (4.95)	14 (4.62)	4.96 (3.20)
Self-help groups	9 (3.64)	4.44 (2.96)	–	–	9 (2.97)	4.44 (2.96)
Consulting	11 (4.45)	4.81 (3.25)	1 (1.79)	7.00 (0.00)	12 (3.96)	5.00 (3.16)
Other	12 (4.86)	13.33 (14.55)	8 (14.29)	25.38 (42.87)	20 (6.60)	18.15 (28.92)
Support services (N of visits)						
Psychiatrist	52 (21.05)	2.85 (3.45)	12 (21.43)	2.67 (2.50)	64 (21.12)	2.81 (3.28)
Psychologist	50 (20.24)	6.76 (8.98)	13 (23.21)	6.15 (6.77)	63 (20.79)	6.63 (8.52)
Counseling/individual therapy	39 (15.79)	23.90 (35.30)	3 (5.36)	2.67 (2.89)	42 (13.86)	22.38 (34.43)
Counseling/group therapy	14 (5.67)	32.36 (23.76)	1 (1.79)	5.00 (0.00)	15 (4.95)	30.53 (23.96)
Family doctor	54 (21.86)	3.00 (3.01)	9 (16.07)	4.11 (4.23)	63 (20.79)	3.16 (3.20)
Therapeutic community - disability	3 (1.21)	11.33 (12.70)	–	–	3 (0.99)	11.33 (12.70)
Therapeutic community (other services)	3 (1.21)	11.00 (11.79)	–	–	3 (0.99)	11.00 (11.79)
Other community - professional team	8 (3.24)	12.56 (11.75)	–	–	8 (2.64)	12.56 (11.75)
Communities - behavioral specialists	3 (1.21)	18.67 (25.48)	1 (1.79)	182.00	4 (1.32)	59.50 (84.27)
Pediatric community/specialized	8 (3.24)	19.5 (40.74)	–	–	8 (2.64)	19.50 (40.74)
Occupational therapist	6 (2.43)	52.00 (37.71)	1 (1.79)	2.00 (0.00)	7 (2.31)	44.85 (39.27)
Speech therapist	26 (10.53)	31.19 (18.00)	–	–	26 (8.58)	31.19 (18.00)
Physiotherapist	2 (0.81)	28.00 (28.28)	2 (3.57)	15.5 (20.51)	4 (1.32)	21.75 (21.42)
Social worker	5 (2.02)	12.00 (20.33)	2 (3.57)	16.50 (13.44)	7 (2.31)	13.28 (17.62)
Home help/home care aide	9 (3.64)	54.22 (24.24)	6 (10.71)	66.33 (44.30)	15 (4.95)	59.07 (32.78)
Social worker/Family support	2 (0.81)	48.00 (0.00)	2 (3.57)	38.00 (19.80)	4 (1.32)	43.00 (12.81)
Adult companion	3 (1.21)	20.67 (9.24)	1 (1.79)	24.00 (0.00)	4 (1.32)	21.50 (7.72)
Day care center	3 (1.21)	47.66 (40.42)	5 (8.93)	140.80 (138.43)	8 (2.64)	105.88 (117.22)
Social club	1 (0.40)	24.00 (0.00)	4 (7.14)	9.75 (8.02)	5 (1.65)	12.60 (9.42)
After-school	8 (3.24)	75.00 (56.07)	–	–	8 (2.64)	75.00 (56.07)
Playful Projects	9 (3.64)	14.56 (13.97)	7 (12.50)	21.57 (14.20)	16 (5.28)	17.63 (14.06)
Protected laboratory	4 (1.62)	52.00 (73.63)	7 (12.50)	50.29 (40.65)	11 (3.63)	50.91 (51.57)
Individual support and inclusion	1 (0.40)	96.00 (0.00)	1 (1.79)	96.00 (0.00)	2 (0.66)	96.00 (0.00) -
One day vacation projects	8 (3.24)	3.75 (1.91)	3 (5.36)	8.00 (6.24)	11 (3.63)	4.91 (3.78)
More than 1 day vacation projects	7 (2.83)	3.29 (5.22)	5 (8.93)	3.40 (1.82)	12 (3.96)	3.33 (4.01) 0.165
Baby-sitter	19 (7.69)	43.32 (38.95)	–	–	19 (6.27)	43.32 (38.95) -
Educator	24 (9.72)	73.96 (69.15)	6 (10.71)	34.50 (45.28)	30 (9.90)	66.07 (66.36)
Other	26 (10.53)	38.73 (35.79)	5 (8.93)	67.80 (59.12)	31 (10.23)	43.42 (40.64)
Residential services (N of days)						
Residential service - children/adolescents	4 (1.62)	104.50 (174.02)	–	–	4 (1.31)	104.50 (174.02)
Residential service - adults	–	–	8 (14.29)	121.00 (105.77)	8 (2.64)	121.00 (105.77)
Foster care (temporary)	1 (0.40)	180.00 (0.00)	–	–	1 (0.33)	180.00 (0.00)
Other	2 (0.81)	93.00 (123.03)	1 (1.79)	180.00 (0.00)	3 (0.99)	122.00 (100.46)
Hospital services (N of accesses)						
Psychiatric Hospital	2 (0.81)	187.50 (251.02)	–	–	2 (0.66)	187.50 (251.02)
Polyclinic psychiatric unit	6 (2.43)	13.50 (15.00)	–	–	6 (1.98)	13.50 (15.00)
General medicine department	5 (2.02)	5.41 (5.50)	1 (1.79)	45.00 (0.00)	6 (1.98)	12.00 (16.91)
Inpatient care in prison/security	–	–	–	–	–	–
Other	3 (1.21)	12.67 (6.43)	2 (3.57)	5.50 (6.64)	5 (1.65)	9.80 (6.80)
Psychiatric outpatient visit	44 (17.81)	9.13 (44.89)	6 (10.71)	2.50 (1.05)	50 (16.50)	8.34 (42.11)
Emergency room	15 (6.07)	1.53 (0.92)	5 (8.93)	1.60 (0.89)	20 (6.60)	1.55 (0.89)

*Percentages were calculated based on the total number of children and adults who responded to each respective question.

In schools or colleges, the professionals with whom autistic individuals interacted the most were support teachers, followed by the primary carers, special classroom assistants (only children), social workers, and others (e.g., behavioral therapists, home assistants, psychologists, teachers of various sports, music therapists), educational psychologists, occupational therapists, speech therapists (only children), and psychomotor therapists/physiotherapists. Fourteen percent of responders answered that none of the above figures had been used, and 5% did not know whether the autistic person had interacted with some of these professionals. Children showed a statistically significant higher mean number of hours per week for responses related to “other” than the listed educational professionals (p=0.020) compared to the adult group. In contrast, adults exhibited a statistically significant higher mean for responses related to “other” than individual and small group lessons compared to the children’s group (p=0.010).

The support/tutoring autistic children and adults received more often included individual lessons at school or college, at home, and lessons in small groups at school. 5% of autistic individuals have faced school removals due to behavioral or other issues (14 out of 271 respondents). Among this group, 3 experienced single instances of exclusion, 5 faced 2 or 3 instances, 4 encountered 4 or 5 instances, and one experienced exclusion more than 5 times within the stated timeframe. Regarding the exclusion duration, 2 individuals were removed for 1 day, 2 individuals for 2 or 3 days, 3 individuals for 2 or 3 days, 3 individuals for 4 to 5 days, and 4 individuals for more than 5 days.

Carers (parent/family member) reported that the most frequently used health or social work services were psychiatric services and marriage counselors.

Fifty-five percent of autistic individuals used support services (5% did not know). Carers also reported that autistic children received one of the following forms of support more often from educators, group therapies, and speech therapists.

Five percent (16 out of 299) of the carers reported that their loved ones received any residential care services (e.g., temporary institutionalization for family problems or support for family members/carers). Six percent (19 out of 298) of the carers reported that their loved ones underwent hospitalization.

Thirty-four percent (99 out of 292) of the carers reported that the autistic person had received outpatient hospital care (i.e., the person has not been admitted overnight but has visited hospitals, clinics, or other centers for diagnosis or treatment). The majority (75%) refrained from utilizing any of the hospital services mentioned while a quarter (25%) remained uncertain about whether the individual had availed themselves of any of these forms of assistance.

Regarding residential services, although a limited number of respondents provided answers (n=16), noteworthy data emerged regarding the duration of stay in various social and health residential services. For children and adolescents, the duration ranged from 3 to 365 days (n=4); similarly, within the social and health residential service for adults ranged from 2 to 288 days (n=8).

Psychiatric hospitals emerged as the most frequented services followed by the psychiatric department of a polyclinic, the general medicine department, and other hospital services. No carers selected inpatient care in prison/security or semi-security settings.

Regarding individuals aged below 18 years, psychiatric outpatient visits were sought 9.13 ± 44.89 times whereas visits to the emergency room occurred an average of 1.53 ± 0.92 times. Concerning adults, psychiatric outpatient visits were utilized an average of 2.50 ± 1.05 times while visits to the emergency room occurred at an average of 1.60 ± 0.89 times.

No statistically significant differences were identified between children and adults in the questions regarding the frequency of accessing services (all p-values ≥ 0.061).

### 3.2 Duration of services’ utilization

**Table 2** shows the results concerning the length of services’ utilization in the last 6 months of the investigated services.

**Table 2:** Total hours of service utilization over the past six months.

	Children (n=247)		Adults (n=56)		Overall (n=303)	
	N (%)	Mean (SD)	N (%)	Mean (SD)	N (%)	Mean (SD)
Services for carers						
Additional visit with family doctor	9 (3.64)	4.28 (9.65)	3 (5.36)	8.67 (13.28)	12 (3.96)	5.38 (10.19)
Family planning	6 (2.43)	3.22 (30.02)	–	–	6 (1.98)	3.22 (3.02)
Social Services	12 (4.86)	1.79 (1.50)	5 (8.93)	4.00 (3.94)	17 (5.61)	2.44 (2.55)
Psychiatric Services	11 (4.45)	1.68 (1.52)	2 (3.57)	2.00 (1.41)	13 (4.29)	1.73 (1.45)
Marriage counselor	3 (1.21)	2.67 (2.89)	–	–	3 (0.99)	2.67 (2.89)
Counseling	10 (4.05)	2.70 (1.70)	2 (3.57)	1.50 (0.71)	12 (3.96)	2.50 (1.62)
Self-help groups	9 (3.64)	5.33 (4.09)	–	–	9 (2.97)	5.33 (4.09)
Consulting	9 (3.64)	3.56 (2.70)	1 (1.79)	1.00 (0.00)	10 (3.30)	3.30 (2.67)
Other	14 (5.67)	23.55 (47.17)	8 (14.29)	10.63 (20.30)	22 (7.26)	18.85 (39.44)
Support services						
Psychiatrist	51 (20.65)	0.96 (0.59)	11 (19.64)	0.86 (0.21)	62 (20.46)	0.94 (0.54)
Psychologist	56 (22.67)	5.09 (26.00)	14 (25.00)	1.11 (0.39)	70 (23.10)	4.29 (23.27)
Counseling/individual therapy	43 (17.41)	1.89 (1.57)	5 (8.93)	23.20(47.44)	48 (15.84)	4.11 (15.39)
Counseling/group therapy	17 (6.88)	1.72 (0.79)	1 (1.79)	2.00 (0.00)	18 (5.94)	1.74 (0.77)
Family doctor	59 (23.89)	0.46 (0.30)	9 (16.07)	0.41 (0.24)	68 (22.44)	0.45 (0.29)
Therapeutic community - disability	5 (2.02)	1.47 (0.51)	–	–	5 (1.65)	1.47 (0.51)
Therapeutic community (other services)	4 (1.62)	1.38 (1.09)	–	–	4 (1.32)	1.38 (1.09)
Other community - professional team	8 (3.24)	2.00 (1.16)	1 (1.79)	0.75 (0.00)	9 (2.97)	1.86 (1.17)
Communities -behavioral specialists	3 (1.21)	1.67 (0.58)	2 (3.57)	15.00 (12.73)	5 (1.65)	7.00 (9.70)
Pediatric community/specialized	10 (4.05)	1.13 (0.50)	–	–	10 (3.30)	1.13 (0.50)
Occupational therapist	7 (2.83)	9.89 (22.10)	1 (1.79)	1.00 (0.00)	8 (2.64)	8.76 (20.71)
Speech therapist	34 (13.77)	21.81 (38.39)	1 (1.79)	0.90 (0.00)	35 (11.55)	21.22 (37.99)
Physiotherapist	3 (1.21)	1.33 (0.58)	2 (3.57)	0.38 (0.18)	5 (1.65)	0.95 (0.67)
Social worker	5 (2.02)	0.80 (0.27)	2 (3.57)	0.78 (0.74)	7 (2.31)	0.79 (0.38)
Home help/home care aide	11 (4.45)	2.56 (1.22)	4 (7.14)	2.69 (1.95)	15 (4.95)	2.59 (1.37)
Social worker/Family support	2 (0.81)	1 (0.00)	2 (3.57)	3.00 (1.41)	4 (1.32)	2.00 (1.41)
Adult companion	3 (1.21)	2.33 (0.58)	1 (1.79)	2.50 (0.00)	4 (1.32)	2.38 (0.48)
Day care center	6 (2.43)	2.75 (1.70)	3 (5.36)	3.00 (1.73)	9 (2.97)	2.83 (1.60)
Social club	2 (0.81)	2.25 (1.06)	3 (5.36)	2.17 (0.76)	5 (1.65)	2.20 (0.76)
After-school	9 (3.64)	2.61 (0.93)	–	–	9 (2.97)	2.61 (0.93)
Playful Projects	11 (4.45)	1.41 (0.74)	6 (10.71)	2.67 (1.37)	17 (5.61)	1.85 (1.14)
Protected laboratory	4 (1.62)	1.75 (0.96)	7 (12.50)	3.14 (1.46)	11 (3.63)	2.64 (1.43)
Individual support and inclusion	1 (0.40)	2.00 (0.00)	1 (1.79)	1.50 (0.00)	2 (0.66)	1.75 (0.35)
One day vacation projects	10 (4.05)	4.90 (2.28)	2 (3.57)	6.00 (2.83)	12 (3.96)	5.08 (2.27)
More than 1 day vacation projects	10 (4.05)	Days: 68 (31.83)	6 (10.71)	161.00 (134.40)	16 (5.28)	102.88 (93.76)
Baby-sitter	23 (9.31)	4.70 (5.71)	1 (1.79)	2.00 (0.00)	24 (7.92)	4.58 (5.61)
Educator	36 (14.57)	2.22 (1.75)	7 (12.50)	2.29 (1.80)	43 (14.19)	2.23 (1.74)
Other (including summer camp 7 days)	27 (10.93)	7.73 (32.04)	5 (8.93)	3.20 (1.79)	32 (10.56)	7.02 (29.40)

Percentages were calculated based on the total number of children and adults who responded to each respective question.

The services for carers used for longer hours were additional visits with the family doctors and self-help groups. Among the support services, psychologists, vacation projects, and babysitters were used for a longer period (measured in hours).

The total hours of support from the psychologist (p=0.050) were higher for children compared to adults, while playful projects (p=0.004), as well as the total days of vacation projects lasting more than 1 day (p=0.050), were higher for adults compared to children. No other statistically significant differences were identified between children and adults in the questions regarding the duration of service utilization (all p-values > 0.070).

### 3.3 Direct payments and service costs

**Table 3** shows the results concerning the direct payment and cost of the services in the last 6 months of the investigated services.

**Table 3:** Direct payments and service costs in euros for the past six months.

	Users directly paying for the service N responders (% of users directly paying)			Service costs in euros N responders; Mean euros/hour (SD)		
	Children	Adults	Overall	Children	Adults	Overall
Educational services						
Educational psychologist	101 (81.18)	8 (75.00)	109 (80.73)	82; 31.87 (14.20)	5; 24.60 (6.58)	87; 31.45 (13.96)
Social worker	32 (3.12)	1 (0.00)	33 (3.30)	1; 20.00 (0.00)		1; 20.00 (0.00)
Special classroom assistant	87 (18.39)	7 (14.29)	94 (18.09)	15; 25.83 (12.78)	1; 20.00(0.00)	16; 25.47 (12.43)
Support teacher	169 (2.96)	7 (14.28)	176 (3.41)	4; 21.50 (7.23)	1; 10.00(0.00)	5; 19.20 (8.11)
Speech therapist	54 (50.00)	1 (100.00)	55 (50.91)	28; 35.36 (9.99)	1; 25.00 (0.00)	29; 35.00 (10.00)
Occupational therapist	17 (35.29)		17 (35.29)	6; 25.83 (11.58)		6; 25.83 (11.58)
Psychomotor therapist/physiotherapist	38 (52.63)	1 (100.00)	39 (53.85)	21; 33.29 (9.44)	1; 35.00 (0.00)	22; 33.36 (9.22)
Primary carer (basic assistant)	37 (2.70)	2 (0.00)	39 (2.56)	3; 17.67 (2.52)		3; 17.67 (2.52)
Other	63 (90.47)	31 (32.29)	94 (71.28)	57; 42.67 (35.22)	10; 79.95 (149.01)	67; 48.24 (65.27)
Individual lessons at home	65 (83.08)	7 (85.71)	72 (83.33)	54; 24.31 (18.57)	6; 17.08 (6.79)	60; 23.59 (17.85)
Individual lessons at school	16 (56.25)	4 (50.00)	20 (55.00)	9; 25.11 (16.97)	2; 21.50 (2.12)	11; 24.45 (15.26)
Small group lessons at school	13 (46.15)	2 (50.00)	15 (46.66)	6; 22.83 (6.79)	1; 18.00 (0.00)	7; 22.14 (6.47)
Other	43 (79.07)	5 (80.00)	48 (79.16)	34; 53.04 (49.81)	3; 37.33 (45.62)	37; 51.76 (49.08)
Services for carers						
Additional visit with family doctor	9 (11.11)	3 (0.00)	12 (8.33)	1; 50.00 (0.00)	–	1; 50.00 (0.00)
Family planning	6 (66.60)	1 (0.00)	7 (57.14)	3; 58.30 (28.43)	–	3; 58.30 (28.43)
Social Services	11 (0.00)	5 (0.00)	16 (0.00)	–	–	–
Psychiatric Services	12 (16.66)	2 (0.00)	14 (14.29)	2; 34.00 (22.63)	–	2; 34.00 (22.63)
Marriage counselor	3 (100.00)	1 (0.00)	4 (75.00)	3; 60.00 (0.00)	–	3; 60 (0.00)
Counseling	11 (63.63)	3 (33.33)	14 (57.14)	7; 114.29 (170.28)	1; 75.00 (0.00)	8; 109.38 (158.26)
Self-help groups	8 (25.00)	1 (0.00)	9 (22.20)	2; 11.50 (4.95)	–	2; 11.50 (4.95)
Consulting	9 (77.78)	2 (50.00)	11 (72.70)	7; 118.57 (96.03)	1; 25.00 (0.00)	8; 106.88 (94.87)
Other	12 (58.33)	9 (66.66)	21 (61.90)	7; 90.43 (116.58)	5; 51.00 (22.47)	12; 74.00 (89.49)
Support services						
Psychiatrist	53 (26.42)	10 (8.30)	63 (23.81)	12; 153.17 (104.96)	1; 90.00 (0.00)	13; 148.31 (102.01)
Psychologist	61 (75.41)	14 (78.57)	75 (76.00)	44; 104.74 (97.35)	11; 73.64 (63.92)	55; 98.52 (91.98)
Counseling/individual therapy	46 (78.26)	5 (100.00)	51 (89.13)	32; 82.14 (116.79)	5; 100.85 (116.28)	37; 84.67 (115.28)
Counseling/group therapy	16 (62.50)	1 (0.00)	17 (58.82)	9; 32.44 (15.45)	–	9; 32.44 (15.45)
Family doctor	57 (10.53)	11 (0.00)	68 (8.82)	6; 110.67 (69.92)	–	6; 110.67 (69.92)
Therapeutic community - disability	6 (66.67)	–	6 (6.67)	3; 60.00 (26.46)	–	3; 60.00 (26.46)
Therapeutic community (other services)	5 (80.00)	–	5 (80.00)	4; 113.75 (98.10)	–	4; 113.75 (98.10)
Other community - professional team	9 (88.89)	1 (0.00)	10 (80.00)	6; 291.93 (640.95)	–	6; 291.93 (640.95)
Communities -behavioral specialists	4 (75.00)	2 (100.00)	6 (83.30)	2; 82.50 (95.46)	2; 80.00 (28.28)	4; 81.25 (57.50)
Pediatric community/specialized	10 (40.00)	–	10 (40.00)	3; 167.33 (203.57)	–	3; 167.33 (203.57)
Occupational therapist	6 (100.00)	1 (0.00)	7 (85.71)	6; 52.33 (18.83)	–	6; 52.33 (18.83)
Speech therapist	32 (62.50)	1 (100.00)	33 (63.64)	19; 41.47 (17.13)	1; 25.00 (0.00)	20; 40.65 (17.08)
Physiotherapist	3 (33.30)	1 (0.00)	4 (25.00)	1; 35.00 (0.00)	–	1; 35.00 (0.00)
Social worker	7 (0.00)	3 (0.00)	10 (0.00)	–	–	–
Home help/home care aide	13 (38.46)	5 (40.00)	18 (38.89)	4; 83.13 (144.80)	2; 18.50 (23.33)	6; 61.58 (117.49)
Social worker/Family support	2 (100.00)	2 (100.00)	4 (100.00)	–	1; 11.00 (0.00)	1; 11.00 (0.00)
Adult companion	3 (100.00)	1 (100.00)	4 (100.00)	2; 150.00 (141.42)	1; 50.00 (0.00)	3; 116.67 (115.47)
Day care center	6 (0.00)	7 (28.57)	13 (15.38)	–	2; 16.25 (12.37)	2; 16.25 (12.37)
Social club	2 (100.00)	4 (75.00)	6 (83.30)	2; 33.00 (24.04)	2; 10.00 (0.00)	4; 21.50 (19.20)
After-school	11 (100.00)	–	11 (100.00)	9; 16.51 (15.11)	–	9; 16.51 (15.11)
Playful Projects	13 (76.93)	8 (75.00)	21 (76.19)	8; 23.69 (31.85)	6; 30.50 (30.49)	14; 26.60 (30.27)
Protected laboratory	5 (60.00)	7 (85.71)	12 (75.00)	3; 19.33 (11.02)	6; 30.33 (22.36)	9; 26.67 (19.31)
Individual support and inclusion	1 (100.00)	1 (100.00)	2 (100.00)	1; 15.00 (0.00)	1; 65.00 (0.00)	2; 40.00 (35.36)
One day vacation projects	10 (90.00)	4 (75.00)	14 (85.71)	7; 31.43 (12.15)	3; 70.00 (52.92)	10; 43.00 (32.68)
More than 1 day vacation projects	10 (70.00)	8 (75.00)	18 (72.20)	6; 262.50 (210.66)	6; 1005.00 (1482.39)	12; 633.75 (1081.38)
Baby-sitter	23 (100.00)	2 (100.00)	25 (100.00)	22; 31.36 (36.38)	2; 16.00 (8.49)	24; 30.08 (35.09)
Educator	37 (72.97)	6 (50.00)	43 (69.77)	25; 34.26 (28.77)	3; 20.00 (5.00)	28; 32.73 (27.53)
Other (including summer camp 7 days)	29 (75.86)	5 (60.00)	34 (73.52)	22; 180.82 (530.57)	3; 70.67 (40.07)	25; 161.15 (488.83)
Residential service						
Residential service - children/adolescents	4 (50.00)	–	4 (50.00)	2; 40.00 (14.14)	–	2; 40.00 (14.14)
Residential service - adults	–	7 (85.70)	7 (85.70)	–	5; 47.34 (34.73)	5; 47.34 (34.73)
Foster care (temporary)	1 (0.00)	–	1 (0.00)	–	–	–
Other	2 (50.00)	1 (0.00)	3 (33.33)	1; 50.00 (00.00)	–	1; 50.00 (50.00)
Hospital services						
Psychiatric Hospital	4 (25.00)	–	4 (25.00)	1; 15000 (0.00)	–	1; 15000 (0.00)
Polyclinic psychiatric unit	6 (16.67)	–	6 (16.67)	–	–	–
General medicine department	5 (20.00)	1 (0.00)	6 (16.67)	–	–	–
Inpatient care in prison/security	–	–	–	–	–	–
Other	3 (0.00)	2 (0.00)	5 (0.00)	–	–	–
Psychiatric outpatient visit	41 (21.95)	5 (0.00)	46 (19.57)	9; 150.78 (73.94)	–	9; 150.78 (73.94)
Emergency room	15 (13.33)	5 (0.00)	20 (10.00)	–	–	–

Educational psychologists (80.73%), other professionals (71.28%), psychomotor therapists/physiotherapists (53.85%), and speech therapists (50.91%) were the professionals most often paid by carers or individuals and the ones paying more per hour. Occupational therapists (35.29%), special classroom assistants (18.09%), support teachers (3.41%), social workers (3.30%), and primary carers (2.56%) were paid less often by the carers or individuals and were the ones paid less per hour.

Carers of autistic children covered the expenses for individual home-based lessons in 83.08% of cases, while for lessons at school or college, the percentage was 56.25%. Similarly, lessons conducted in small groups were funded by carers in 46.15% of instances. Carers of autistic children paid for 79.07% of the other services. Individual lessons at school were reported as the most expensive, followed by those at home), and in small groups. Other educational services were reported to be also quite expensive.

Regarding autistic adults, in 85.71% of cases, the carers or the adults themselves financed individual home-based lessons, whereas the rate for lessons at school or college stood at 50.00%. Similarly, lessons conducted in small groups were financed by carers in 50.00% of instances. As for other services, 80.00% were covered by carers or individuals. Individual lessons at school were reported as the most expensive, followed by those in small groups and at home. Other educational services were reported to be also quite expensive.

The services for carers most frequently paid by them were marriage counselors (75.00%) and consulting (72.70%). Counseling and consulting were reported as the most expensive services.

Among the support services, counseling/individual therapists (89.13%) and psychologists (76.00%) were the professionals most frequently paid by the carers of autistic children. Carers of autistic children reported that psychiatrists and psychologists were reported as the most expensive. A limited number of responses, ranging from 1 to 14 for each question, were collected for adults. Given the modest sample size, the observed results are not substantial enough for comprehensive reporting. Nevertheless, these findings have been tabulated in **Table 3**.

Regarding costs of residential services, the most financially demanding service was the social and health residential service for adults followed by the social and health residential service for children and adolescents.

Regarding costs of hospital services, 25.00% reported covering the expenses for psychiatric hospital, and 19.57% for psychiatric outpatient visits. Concerning adults, no carers or individuals notably reported covering the expenses for these hospital services.

No statistically significant differences were identified between children and adults in the questions regarding the direct payments and service costs (all p-values > 0.080).

### 3.6 Psychopharmacological therapy

Twenty percent (55 out of 281) of carers reported that the autistic person took any psychopharmacological medication in the past 6 months (i.e., for associated symptoms or ASD-related conditions). Of those 55, over half (58%, n=32) were children. Among both children and adults, the most commonly used drugs were psycholeptics (73%, n=40), followed by antiepileptics (16%, n=9).

## 4 Discussion

This work explores the experiences of carers of autistic individuals, shedding light on the demographics, caregiving responsibilities, and utilization of support services. The survey collected responses from 303 carers and focused on autistic individuals, ranging from children to adults. The majority (82%) of the cared-for autistic individuals were children under 18, primarily males (82%). Most of them lived at home with their parents (97%). Carers reported a substantial caregiving load, spending an average of 58.84 hours per week caring for autistic individuals. Many carers (49%) were employed, working an average of 32.23 hours per week. Notably, 82% of carers reported making absences from their work or place of study in the past 6 months to care for autistic individuals, with an average of 15.56 days of absence.

More than half (55%) of the autistic individuals received some support from residential care services and outpatient hospital care. Carers play a crucial role in the lives of autistic individuals, contributing significantly to their overall well-being. The results of this study underline the challenges faced by carers of autistic individuals, with a substantial caregiving responsibility that often results in work or study absences.

### 4.1 Interactions with professionals and utilization of educational support for autistic individuals

The study reveals that, within school or college environments, not surprisingly, the support teacher is the professional with whom autistic individuals interact the most frequently. In Italy, support teachers are professionals specialized in assisting students with disabilities or special educational needs within the school system. This service is made possible by Law 104/92 (10), which provides support measures for students with disabilities. The number of hours of support provided by a support teacher to a child with disabilities or special educational needs can vary widely depending on the student’s individual needs, decisions made by the school, and local regulations. Hours of support are generally established through the student’s Individualized Education Plan (*Piano Educativo Individualizzato*, PEI), which is developed in collaboration between the school, teachers, and parents. The PEI takes into account the student’s specific needs and establishes the educational goals and support strategies needed. This underlines the fundamental role played by support teachers in facilitating the educational experiences of autistic students. Policymakers should prioritize providing comprehensive training for support teachers, focusing on understanding the specific needs of autistic individuals, using effective learning methods tailored to their requirements, and using strategies that foster improved socialization with their peers.

Primary carers and special classroom assistants (for children) are also prominently involved in the interactions, highlighting the importance of these figures in providing personalized care and support to autistic individuals. Social workers, educational psychologists, and other professionals were also part of the support network, contributing to a multidisciplinary approach to ASD care within educational settings.

Individual lessons, whether at school or home, were reported as the most common forms of support for children and adults. This suggests the need for tailored, one-on-one learning experiences to address the unique learning styles and needs of autistic individuals. Additionally, lessons are provided in small groups, emphasizing the importance of balanced peer interactions and social learning opportunities. Notably, other educational services are widely employed, suggesting a diverse range of interventions and strategies employed to cater to the individualized needs of autistic individuals.

The study highlights a concerning aspect of the educational experiences of autistic individuals, with 5% of respondents reporting that some individuals were distanced from school due to behavioral problems. The frequency of exclusion and the corresponding days underscore the challenges educators and carers face in managing and supporting the behavior of autistic individuals. This aspect warrants further investigation into the triggers of such behaviors, the support systems in place to address them, and the impact of exclusion on the overall well-being of the individuals involved.

The financial dimension of ASD care and support is an essential aspect explored in the study. However, even with recommendations urging the incorporation of family costs and burdens (11), there remains a lack of understanding concerning the economic consequences of ASD treatments, as emphasized by several studies (12–15). Carers and individuals more frequently reported to pay for some interventions. In the present survey, educational psychologists, speech therapists, and psychomotor therapists/physiotherapists are the most paid professionals. Despite the contributions these professionals make to the development of autistic individuals, carers frequently find themselves resorting to privately paid services. The results emphasize the importance of fostering collaborations between educators, carers, and professionals to provide comprehensive and effective support for autistic individuals.

### 4.2 Utilization of health and social services by carers of autistic individuals

The study reveals that psychiatric services and marriage counseling were the two most frequently used health or social work services by carers during the past 6 months. The engagement with psychiatric services underscores the complexity of caring for autistic individuals, as it suggests a need for specialized mental health support to address both the individuals’ needs and the carers’ well-being. The prominence of marriage counseling highlights the recognition of caregiving’s impact on familial relationships and the importance of seeking professional guidance in navigating these challenges.

Two services, additional visits with the family doctor and participation in self-help groups, were used for longer durations. The engagement with the family doctor suggests a multifaceted approach to addressing the health and well-being of the autistic individual and the carer. The use of self-help groups implies the significance of peer support and shared experiences among carers, offering emotional solace, advice, and coping strategies within a community of individuals facing similar challenges.

Marriage counseling and consulting services were reported as the most frequently paid for by carers, reflecting the importance of professional guidance and support, often accessible through private service avenues. Interestingly, counseling and consulting services were also reported as the most expensive, emphasizing the financial commitment required to access these specialized resources. These findings underscore the need for affordable and accessible support services that cater specifically to the unique challenges of caring for autistic individuals.

The utilization of psychiatric services and marriage counseling indicates the multidimensional needs of both autistic individuals and their carers. Policymakers and service providers should consider these insights to tailor support systems that address mental health and familial dynamics. Research suggests that children from families with lower overall functioning are less likely to experience the same positive outcomes from comparable treatment interventions (16, 17). Therapeutic efforts should prioritize enhancing parental perspectives concerning their child’s diagnosis, emotional parenting experience, marital contentment, and the role of siblings (18).

The extended utilization of additional family doctor visits and self-help groups speaks to the need for care approaches that encompass medical, psychological, and emotional aspects. The high costs associated with counseling and consulting services signal the importance of considering financial assistance options for carers to ensure equitable access to essential resources.

### 4.3 Support utilization for autistic individuals

The study reveals that 55% of autistic individuals received support in the past 6 months. These results highlight the importance of establishing a support network for autistic individuals, underscoring the need to address their unique needs and challenges. Among the most frequently accessed forms of support for autistic children were educators, group therapy, and speech therapists. These findings suggest the value of educational and therapeutic interventions in promoting the development and well-being of autistic children.

Certain support services were reported to be used for longer periods, implying their sustained impact and contribution to the well-being of autistic individuals. Psychologists, vacation projects, and babysitters were utilized for more extended hours. The prolonged engagement with psychologists underscores their vital role in addressing the psychological and behavioral needs of autistic children. The involvement of vacation projects and babysitters further indicates the significance of providing respite and recreational opportunities for individuals and their carers.

Counseling/individual therapists and psychologists were the professionals most frequently paid by carers of autistic children. The variations in payment suggest the specialized nature of these services and the financial commitment required to access them. The higher cost associated with psychiatrists and psychologists highlights the need for adequate financial resources to provide comprehensive mental health care.

### 4.4 Residential care services for autistic individuals

The study reveals that a relatively small percentage (5%) of autistic individuals received residential care services in the last 6 months. Although the number of responders was limited, the findings still offer valuable insights into the role of residential care in the spectrum of support services available for autistic individuals. This highlights the significance of addressing the diverse needs of this population and offering options that cater to their specific circumstances.

For children and adolescents, the number of days spent in social and health residential services ranged widely, suggesting a high variability in the care needs and circumstances of the individuals accessing these services. Similarly, the costs associated with residential care varied, with social and health residential services for adults reported as the most expensive, followed by those for children and adolescents. These variations could be attributed to the complexity of care, the nature of services provided, and the level of individualized attention required.

It is noteworthy that, in some cases, carers or individuals themselves were responsible for covering the costs of residential care services. This financial responsibility adds to the overall load of caregiving, underscoring the need for accessible and affordable residential care options. The difference in payment patterns may be due to healthcare policies, public funding availability, and each case’s specific circumstances. Policymakers should recognize the need for accessible, high-quality residential care options that cater to various age groups and individual needs. Furthermore, financial assistance and support programs may be necessary to alleviate the financial strain of accessing these services.

### 4.5 Hospital services for autistic individuals

The study indicates that a minority (6%) of autistic individuals experienced hospitalizations in the last 6 months, while a larger percentage (34%) received outpatient hospital care. The prevalence of outpatient care suggests the importance of strengthening non-inpatient services in ASD care. Additionally, a noteworthy percentage of carers (25%) did not know whether their loved ones received hospital support, underscoring the communication gap between carers, individuals, and medical professionals.

The study highlights that psychiatric hospital services were the most frequently used type of hospital care, followed by the psychiatric department of a polyclinic and general medicine departments. These findings signal the significance of addressing mental health and general medical concerns in the care of autistic individuals. Notably, some carers reported using other hospital services for a relatively short duration, indicating varied health needs.

Among the carers of children and adolescents, a portion reported to pay for psychiatric outpatient visits, indicating a financial commitment to accessing mental health care. In contrast, no carers or individuals paid for the hospital services used by adults.

The findings emphasize the need for comprehensive medical care that addresses the mental and general health needs of autistic individuals. Policymakers should consider the prevalence of outpatient care, recognizing its significance in the overall healthcare landscape for autistic individuals.

The lack of statistically significant differences between children and adults in questions related to service utilization duration, service access frequency, and direct payments and service costs) suggests similarity in patterns of service use and access between the two groups. However, it is important to note that the absence of statistical significance does not necessarily imply the absence of substantive or practically meaningful differences. Further investigations may be useful to explore more deeply any variations or trends in service utilization and access behaviors between children and adults with autism.

### 4.6 Psychopharmacological therapy

The study highlights that a minor proportion (20%) of carers reported that their autistic loved ones took psychopharmacological therapy in the past 6 months.

Although the population taking medications is the minority, the majority (58%) were children. This age distribution may reflect the early need to manage ASD-related symptoms.

As previously reported (19), the most used psychopharmacological medications for both autistic children and adults were psycholeptics (73%), followed by antiepileptics (16%). Medications are frequently used to address associated symptoms (20), such as hyperactivity, inattention, impulsivity, agitation, anxiety, obsessive-compulsive symptoms, and seizures. This reflects the fact that autistic individuals often have a complex array of psychiatric, neurological, and medical co-occurring conditions that extend beyond the core features of ASD itself (21–24). Further, healthcare providers should consider a comprehensive care approach integrating both behavioral and pharmacological interventions, along with monitoring side effects and the number of prescriptions to the same individuals (25).

The high utilization of psycholeptics highlights the prominence of addressing emotional and behavioral difficulties in autistic individuals. However, it is important to recognize that medication use is just one facet of comprehensive care for autistic individuals. Medications should be considered within a broader care plan that prioritizes the individual’s strengths and challenges.

### 4.7 Limitations and perspectives for future studies

Acknowledging the study’s potential limitations, such as the relatively small sample size (303 respondents who sometimes did not complete all the questions included in the survey) and potential biases in self-reporting, is essential. In certain survey questions, the limited sample size prevented the calculation of statistical differences between the mean of the children’s group and the mean of the adult group, highlighting the need for verification in studies with more appropriate sample sizes. Indeed, it is crucial to note that we were unable to provide data on the transitional age group due to the limited number of participants in this age range (n=19 individuals aged 16-18 years). These limitations could impact the finding’s generalizability and should be considered when interpreting the results. Furthermore, the survey did not investigate whether individuals with autism received economic support, such as disability pensions or attendance allowances, and it did not collect some other crucial demographic data (e.g., subjects’ level of functioning according to DSM-5 and age at diagnosis/time elapsed from diagnosis). These crucial aspects should be explored in future studies. Furthermore, future research should give priority to examining the handling of behavioral emergencies and hospitalizations, exploring whether they stem from behavioral or medical issues and identifying the exact health need that resulted in hospitalization. However, these findings are important to understand the complex caregiving landscape, the diverse range of support services available in Italy, and the critical role of professionals in addressing the needs of autistic individuals. Future studies should seek to extend this survey longitudinally, thereby expanding the participant pool. This approach will enhance the robustness of conclusions drawn regarding autism services in Italy, and it will also facilitate the monitoring of changes in service costs over the years. In addition, subsequent research may bring out the positive outcome effect of investments activated after the end of the survey.

### 4.8 Conclusions

In conclusion, this data highlights the multifaceted nature of care provision, the financial implications of seeking specialized support, and the importance of mental health and relational well-being for carers and those they care for. The prevalence of support utilization indicates the need for a comprehensive network of services to cater to the diverse needs of autistic individuals. The involvement of various professionals underscores the multidisciplinary nature of ASD care and emphasizes the significant financial investment that carers make to provide essential services for their loved ones. Caring for autistic individuals can be costly, but with the right services and support, it is possible to minimize the impact of ASD on an individual’s financial well-being. More research is needed to understand better the costs and services associated with ASD and to develop effective strategies for reducing these costs and improving the quality of life for autistic individuals and their families.

## Data availability statement

The original contributions presented in the study are included in the article/**Supplementary Materials**, further inquiries can be directed to the corresponding author/s.

## Ethics statement

The studies involving humans were approved by National ethics committee for clinical trials of public research bodies (EPR) and other national public institutions (CEN) at the Istituto Superiore di Sanità (ISS). The studies were conducted in accordance with the local legislation and institutional requirements. The participants provided their written informed consent to participate in this study.

## Author contributions

MM: Conceptualization, Data curation, Investigation, Methodology, Writing – original draft, Writing – review & editing. FF: Conceptualization, Data curation, Investigation, Methodology, Writing – review & editing. TS: Data curation, Formal analysis, Writing – review & editing. GR: Funding acquisition, Validation, Writing – review & editing. MS: Conceptualization, Funding acquisition, Investigation, Project administration, Resources, Validation, Writing – review & editing.
