# Supplementary material for: Access and cost of services for autistic children and adults in Italy: a carers’ perspective
Source: Front Psychiatry. 2024 Mar 12;15:1299473. doi: 10.3389/fpsyt.2024.1299473 (PMC10963481; doi:10.3389/fpsyt.2024.1299473)
Supplement: Supplementary file 1 [file DataSheet_1.docx]

Supplementary Material

Access and cost of services for autistic children and adults in Italy: A carers’ prospective

Martina Micai^1^, Francesca Fulceri^1^, Tommaso Salvitti^1^, Giovanna Romano^2^, Maria Luisa Scattoni^1*^

*** Correspondence:** Corresponding Author: marialuisa.scattoni@iss.it

# Supplementary Data. Italian version of the questionnaire on the use of resources related to Autism Spectrum Disorder

INTRODUZIONE

La ringraziamo per aver deciso di dedicare del tempo alla compilazione di questo questionario.

Il questionario raccoglie le **informazioni sull’accesso ai servizi educativi, all’assistenza sanitaria, ai servizi sociali e ad altri servizi correlati delle persone con autismo di tutte le età.**

Potrà compilare il questionario se è una persona con autismo, un genitore o l’assistente/caregiver di una persona con autismo a cui lui/lei ha dato il consenso per rispondere al suo posto. Alla fine del questionario c’è una sezione con domande specifiche per i genitori, i familiari o gli assistenti/caregivers.

Il questionario è anonimo e impiegherà circa 20-30 minuti per completarlo. Potrà salvare le sue risposte e continuare in qualsiasi momento. Le domande contrassegnate con un asterisco (*) sono obbligatorie.

**IDENTIFICAZIONE**

La preghiamo di rispondere alle seguenti domande solo una volta. Se è un genitore o l’assistente/caregiver di una persona con autismo, potrà completare il questionario al suo posto. Se ha il permesso di rispondere al questionario per più di una persona con autismo, la preghiamo di completare il questionario per ogni persona separatamente. Per esempio, se vuole rispondere al questionario per 2 persone con autismo completi il questionario per due volte. Per favore, non risponda al questionario per la stessa persona più di una volta.

1. Chi sta rispondendo al questionario? (*)

| Io sono una persona con autismo |
| --- |
| Io sono il genitore di una persona con disturbo dello spettro autistico |
| Io sono un familiare (non un genitore) di una persona con disturbo dello spettro autistico  Io sono il/la consorte o il compagno/a di una persona con disturbo dello spettro autistico |
| Io sono una persona che si prende cura di una persona con disturbo dello spettro autistico (non un familiare, consorte o compagno/a) |

**DATI DEMOGRAFICI DELLA PERSONA CON DISTURBO DELLO SPETTRO AUTISTICO**

1. Età della persona con autismo (in anni): _______ (*)
2. Genere della persona con autismo: (*)

| Maschio | ⭘ |
| --- | --- |
| Femmina | ⭘ |
| Altro/Nessuna risposta | ⭘ |

1. Qual’è la nazionalità della persona con autismo?

- Italiana ⭘
- Doppia nazionalità, italiana e altro (per favore, specifichi quale): ____________________
- Altra nazionalità (per favore, specifichi quale): ___________________________________

1. Paese di residenza della persona con autismo (*)

| Austria | ⭘ |
| --- | --- |
| Belgio | ⭘ |
| Bulgaria | ⭘ |
| Croazia | ⭘ |
| Cipro | ⭘ |
| Republica Ceca | ⭘ |
| Danimarca | ⭘ |
| Estonia | ⭘ |
| Finlandia | ⭘ |
| Francia | ⭘ |
| Germania | ⭘ |
| Grecia | ⭘ |
| Ungheria | ⭘ |
| Islanda | ⭘ |
| Irlanda | ⭘ |
| Italia | ⭘ |
| Lettonia | ⭘ |
| Lituania | ⭘ |
| Lussemburgo | ⭘ |
| Malta | ⭘ |
| Olanda | ⭘ |
| Norvegia | ⭘ |
| Polonia | ⭘ |
| Portogallo | ⭘ |
| Romania | ⭘ |
| Slovacchia | ⭘ |
| Slovenia | ⭘ |
| Spagna | ⭘ |
| Svezia | ⭘ |
| Regno Unito | ⭘ |
| Altro (per favore specificare): | ⭘ |
|  | |

1. Regione dell’attuale residenza della persona con autismo:

DIAGNOSTICA DELL’ AUTISMO

1. Per favore, specifichi il sottotipo di disturbo dello spettro autistico:

| Disturbo Autistico | ⭘ |
| --- | --- |
| Sindrome di Asperger | ⭘ |
| Disturbi generalizzati dello sviluppo non altrimenti specificati (DGS-NAS) | ⭘ |
| [Disturbo disintegrativo dell’infanzia](http://en.wikipedia.org/wiki/Childhood_disintegrative_disorder) | ⭘ |
| Altro (per favore specificare) | ⭘ |
|  |  |

1. Per favore, specifichi altre condizioni della persona con autismo mai diagnosticate da un medico (per favore segni tutte le voci pertinenti).

| Non so | ⃝ |
| --- | --- |
| Epilessia | ⃝ |
| Disturbo del sonno | ⃝ |
| Disturbi gastrointestinali | ⃝ |
| Ansia | ⃝ |
| Depressione | ⃝ |
| Schizofrenia/Psicosi | ⃝ |
| Disturbo bipolare | ⃝ |
| [Disturbo da deficit di attenzione e iperattività](http://www.nimh.nih.gov/health/topics/attention-deficit-hyperactivity-disorder-adhd/) (ADHD) | ⃝ |
| Disturbo ossessivo-compulsivo | ⃝ |
| Sindrome di Tourette/Tics | ⃝ |
| Sindrome di Down | ⃝ |
| Disabilità Intellettiva | ⃝ |
| Altro (per favore specificare) | ⃝ |
|  |  |

Quale punteggio ha raggiunto la persona con autismo nel più recente test d’intelligenza effettuato (QI)?

| Non so/Nessuna risposta | ⭘ |
| --- | --- |

SISTEMAZIONE ABITATIVA DELLA PERSONA CON AUTISMO

1. Dove sta vivendo attualmente la persona con autismo? (per favore, segni tutte le voci pertinenti)

| In casa con i genitori o i parenti | ⭘ |
| --- | --- |
| In casa con amici/coinquilini | ⭘ |
| In casa con un compagno/a | ⭘ |
| In casa da solo | ⭘ |
| In un bed&breakfast/hotel | ⭘ |
| In un ostello | ⭘ |
| In un affidamento formale | ⭘ |
| In una casa protetta | ⭘ |
| In un istituto scolastico residenziale | ⭘ |
| In un centro residenziale | ⭘ |
| In un ospedale/centro o unità di salute mentale | ⭘ |
| In prigione/carcere minorile/unità di sicurezza | ⭘ |
| Altro (per favore specificare) | ⭘ |
|  |  |

**STATO D’IMPIEGO DELLA PERSONA CON AUTISMO**

Questa sezione va completata solo per le persone di età superiore ai 16 anni che hanno lasciato la scuola.

1. Qual è lo stato attuale di occupazione della persona con autismo? (per favore segni tutte le voci pertinenti).

| Impiego (stipendiato, includendo apprendistato/internato o altra formazione) | ⭘ |
| --- | --- |
| Impiego (non stipendiato, includendo apprendistato/internato o altra formazione o lavoro volontario) | ⭘ |
| Impiego protetto | ⭘ |
| Disoccupato – ma disposto a lavorare | ⭘ |
| Disoccupato – e non disposto a lavorare | ⭘ |
| Pensionato – e non svolge altra attività lavorativa | ⭘ |
| Casalinga/marito – e non svolge altra attività lavorativa | ⭘ |
| Studente a tempo pieno – e non svolge altra attiviità lavorativa | ⭘ |
| Altro (per favore specificare) | ⭘ |
|  |  |

1. Se impiegato (retribuito, includendo l’impiego protetto), quante ore per settimana la persona con autismo lavora in un’impiego retribuito?

|  |
| --- |

1. Se impiegato (non retribuito), quante ore per settimana la persona con autismo lavora in un’impiego non retribuito?

|  |
| --- |

1. Se impiegato (retribuito, includendo lavoro protetto), la persona con autismo ha fatto assenze dal lavoro negli ultimo 6 mesi a causa dell’autismo?

| Non so | ⭘ |
| --- | --- |
| No | ⭘ |
| Si | ⭘ |
| 14b. Se si, per favore specifichi quanti giorni negli ultimi 6 mesi | |
|  | |

**SERVIZI EDUCATIVI A CUI LA PERSONA CON AUTISMO HA AVUTO ACCESSO NEGLI ULTIMI 6 MESI**

Questa sezione va completata solo per le persone con autismo che non hanno lasciato la scuola.

1. Per favore segni tutto quello che ha frequentato negli ultimi 6 mesi

| Niente | ⭘ |
| --- | --- |
| Scuola tradizionale/statale | ⭘ |
| Scuola superiore/Istituto tecnico | ⭘ |
| Università | ⭘ |
| Unità speciali interne alla scuola | ⭘ |
| Scuola speciale (in generale) | ⭘ |
| Scuola speciale per l’autismo | ⭘ |
| Scuola speciale residenziale (in generale) - 38 settimane (durante l’anno scolastico) | ⭘ |
| Scuola speciale residenziale (in generale) - 52 settimane (l’intero anno) | ⭘ |
| Scuola speciale residenziale per l’autismo - 38 settimane (durante l’anno scolastico) | ⭘ |
| Scuola speciale residenziale per l’autismo - 52 settimane (l’intero anno) | ⭘ |
| Scuola domiciliare (come alternativa alla scuola) | ⭘ |
| Altro (per favore specificare) | ⭘ |
|  |  |
|  |  |

1. Quali professionisti, che lavorano in una scuola o università, la persona con autismo ha visto negli ultimi 6 mesi? Se questi professionisti sono pagati per uno di questi servizi diretti (sia con fondi personali o altro) per favore ne indichi il costo, se noto.

|  | Ore per settimana | Pagato direttamente dal caregiver/persona con autismo (Si/No) | Se si, indicare il costo per ora (euro) |
| --- | --- | --- | --- |
| Psicologo dell’educazione |  |  |  |
| Assistente sociale |  |  |  |
| Assistente specialistico in classe |  |  |  |
| Insegnante di sostegno |  |  |  |
| Logopedista (a scuola/università) |  |  |  |
| Terapista occupazionale (a scuola/università) |  |  |  |
| Psicomotricista/Fisioterapista (a scuola/università) |  |  |  |
| Assistente di base (a scuola/università) |  |  |  |
| 16b. Se altro, per favore specificare il tipo di servizio: |  |  |  |
| Nome del servizio | Ore per settimana | Pagato direttamente dal caregiver/persona con autismo (Si/No) | Se si, indicare il costo per ora (euro) |
|  |  |  |  |
|  |  |  |  |
|  |  |  |  |
|  |  |  |  |

1. Se nessuna delle figure di cui sopra è stata utilizata negli ultimi 6 mesi o non lo sa, per favore segni qui:

Nesuna delle figure di cui sopra è stata utilizzata negli ultimi 6 mesi ⭘

Non so se la persona con autismo ha visto alcuni di questi professionisti ⭘

**SUPPORTO/TUTORAGGIO DELLA PERSONA CON AUTISMO**

1. La persona con autismo ha ricevuto qualche forma di supporto negli ultimi 6 mesi?

| No | ⭘ |
| --- | --- |
| Si | ⭘ |

1. Se si, la persona con spettro autistico che tipo di supporto/tutoraggio ha ricevuto negli ultimi 6 mesi? Se chi si occupa di lui o l’individuo stesso paga uno di questi servizi (sia con fondi personali sia sostenuti con un sussidio o indennità) si prega, se noto, di indicarne il costo.

|  | Ore per settimana | Pagato direttamente dal caregiver/dalla persona con autismo  (Si/No) | Se si, indicare il costo per ora (euro) |
| --- | --- | --- | --- |
| Lezioni individuali a casa |  |  |  |
| Lezioni individuali (es. Scuola, università) |  |  |  |
| Lezioni in piccolo gruppo (es. Scuola/università) |  |  |  |
| 19b. Se altro, per favore specificare: |  |  |  |
| Nome del servizio | Ore per settimana | Pagato direttamente dal caregiver/dalla persona con autismo  (Si/No) | Se si, indicare il costo per ora (euro) |
|  |  |  |  |
|  |  |  |  |
|  |  |  |  |
|  |  |  |  |

1. Negli ultimi 6 mesi la persona nello spettro autistico è stata allontanata da scuola (o da un altro istituto scolastico) a causa di comportamenti problema?

| No | ⭘ |
| --- | --- |
| Si | ⭘ |

1. Se la risposta alla precedente domanda è sì, si prega di specificare il numero di volte, che la persona con autismo è stata esclusa e la durata di tempo in totale per cui è stata allontanata negli ultimi 6 mesi.

| Numero di volte: |  |
| --- | --- |
| Numero di giorni in totale: |  |

**MISURE SULLA SALUTE E SUI SERVIZI DI ASSISTENZA SOCIALE**

1. La persona nello spettro autistico ha ricevuto un qualche servizio di assistenza residenziale negli ultimi 6 mesi? (ad esempio, istituzionalizzazione temporanea della persona con autismo per fornire sostegno ai familiari/caregivers).

| No | ⭘ |
| --- | --- |
| Si | ⭘ |

1. Se si, si prega di fornire informazioni in tutte le voci pertinenti

|  | Numero di giorni trascorsi nel servizio residenziale negli ultimi 6 mesi | Pagato direttamente dal caregiver/dalla persona con autismo  (Si/No) | Se si, indicare il costo per giorno (euro) |
| --- | --- | --- | --- |
| Servizio residenziale socio-sanitario per bambini/adolescenti |  |  |  |
| Servizio residenziale socio-sanitario per adulti |  |  |  |
| Affidamento (temporaneo) |  |  |  |
| 23b. Se diverso, si prega di indicare il tipo di struttura: |  |  |  |
| Nome della struttura | Numero di giorni trascorsi nel servizio residenziale socio-sanitario negli ultimi 6 mesi | Pagato direttamente dal caregiver/dalla persona con autismo  (Si/No) | Se si, indicare il costo per giorno (euro) |
|  |  |  |  |
|  |  |  |  |
|  |  |  |  |

1. La persona nello spettro autistico ha subito un ricovero ospedaliero negli ultimi 6 mesi? (cure ricevute durante la sua permanenza per una o più notti in un ospedale)

| No | ⭘ |
| --- | --- |
| Si | ⭘ |

1. Se la risposta alla precedente domanda è sì, si prega di fornire informazioni in tutte le voci pertinenti

|  | Numero di giorni trascorsi negli ultimi 6 mesi | Pagato direttamente dal caregiver/dalla persona con autismo  (Si/No) | Se si, indicare il costo per giorno (euro) |
| --- | --- | --- | --- |
| Ospedale Psichiatrico |  |  |  |
| Reparto psichiatrico di un policlinico |  |  |  |
| Reparto di medicina generale |  |  |  |
| Cure ospedaliere in carcere/in regime di sicurezza o di semi-sicurezza |  |  |  |
| 25b. Se altro, specificare: |  |  |  |
| Tipo di centro | Numero di giorni trascorsi negli ultimi 6 mesi | Pagato direttamente dal caregiver/dalla persona con autismo  (Si/No) | Se si, indicare il costo per giorno (euro) |
|  |  |  |  |
|  |  |  |  |
|  |  |  |  |

1. La persona nello spettro autistico ha ricevuto cure ospedaliere ambulatoriali negli ultimi 6 mesi? (cioè, la persona non è stata ricoverata in ospedale durante la notte ma ha effettuato visite in ospedale, cliniche o in altri centri per la diagnosi o il trattamento)

| No | ⭘ |
| --- | --- |
| Si | ⭘ |

1. Se la risposta alla precedente domanda è sì, si prega di fornire informazioni in tutte le voci pertinenti

|  | Numero di accessi al servizio negli ultimi 6 mesi | Pagato direttamente dal caregiver/dalla persona con autismo  (Si/No) | Se si, indicare il costo per visita (euro) |
| --- | --- | --- | --- |
| visita ambulatoriale psichiatrica |  |  |  |
| Pronto soccorso |  |  |  |
| 27b. Se diverso dalla visita ambulatoriale in ospedale (escluso il pronto soccorso), per favore specificare: |  |  |  |
| Tipo di servizio | Numero di accessi al servizio negli ultimi 6 mesi | Pagato direttamente dal caregiver/dalla persona con autismo  (Si/No) | Se si, indicare il costo per visita (euro) |
|  |  |  |  |
|  |  |  |  |
|  |  |  |  |
|  |  |  |  |
|  |  |  |  |

1. Si prega di specificare se la persona nello spettro autistico ha ricevuto una delle seguenti forme di supporto negli ultimi 6 mesi, completando le sezioni pertinenti nella tabella sottostante. Si prega di non inserire i servizi ricevuti a scuola / università o in una struttura residenziale in cui l'individuo vive. Se chi si occupa di lui o l’individuo stesso paga uno di questi servizi (sia con fondi personali sia se sostenuti con un sussidio o indennità) si prega, se noto, di indicarne il costo.

|  | Visite negli ultimi 6 mesi | Durata media della visita (se nota) | Pagato da chi si occupa di lui/dalla persona stessa  (Si/No) | Se si, qual è il costo per visita? (euro) |
| --- | --- | --- | --- | --- |
| Psichiatra |  |  |  |  |
| Psicologo |  |  |  |  |
| Counselling/terapia individuale |  |  |  |  |
| Counselling/terapia di gruppo |  |  |  |  |
| Medico di famiglia |  |  |  |  |
| Comunità terapeutica specializzata in disabilità |  |  |  |  |
| Comunità terapeutica (altri servizi) |  |  |  |  |
| Altra comunità specializzata in disabilità con team professionale. |  |  |  |  |
| Comunità con personale specializzato in comportamenti problema |  |  |  |  |
| Comunità pediatrica/centro specializzato sull’età evolutiva |  |  |  |  |
| Terapista occupazionale |  |  |  |  |
| Logopedista |  |  |  |  |
| Fisioterapista |  |  |  |  |
| Assistente sociale |  |  |  |  |
| Aiuto domiciliare/assistente domiciliare |  |  |  |  |
| Operatore sociale/ Supporto familiare |  |  |  |  |
| Compagno adulto |  |  |  |  |
| Centro diurno |  |  |  |  |
| Social club |  |  |  |  |
| Doposcuola |  |  |  |  |
| Progetti Ludici |  |  |  |  |
| Laboratorio protetto |  |  |  |  |
| Supporto e inserimento individuale |  |  |  |  |
| Progetti Vacanze (1 giorno; simile ai social clubs, progetti ludici o attività giornaliere) |  | (numero di ore) |  |  |
| Progetti vacanze (più di 1 giorno; simile ad un viaggio vacanze) |  | (numero di giorni) |  |  |
| Baby-sitter |  |  |  |  |
| Altro (specificare di che tipo) | Visite negli ultimi 6 mesi | Durata media delle visite (se noto) | Pagato da chi si occupa di lui/dalla persona stessa  (Si/No) | Se si, qual è il costo per visita?(euro) |
|  |  |  |  |  |
|  |  |  |  |  |
|  |  |  |  |  |

1. Se nessuno dei servizi sopraelencati è stato usato negli ultimi 6 mesi, o non è noto, per favore segni qui:

Nessuno tra i servizi elencati sopra è stato usato ⭘

Non so se la persona con autismo ha ricevuto qualcuno di questi supporti ⭘

1. La persona nello spettro autistico ha preso dei farmaci connessi all’autismo negli ultimi 6 mesi? (cioè, per i sintomi dell’autimo o per condizioni legate all’autismo)

| No | ⭘ |
| --- | --- |
| Si | ⭘ |

1. Se si, si prega di fornire le seguenti informazioni:

| Nome del farmaco | Posologia (se noto) | Frequenza di somministrazione | Per quanto tempo la persona nello spettro autistico ha preso questo farmaco negli ultimi 6 mesi? (Si prega di specificare il numero di settimane) |
| --- | --- | --- | --- |
|  |  |  |  |
|  |  |  |  |
|  |  |  |  |
|  |  |  |  |
|  |  |  |  |

IMPATTO SUI GENITORI/FAMILIARI/CAREGIVERS

Per favore completi questa sezione, solo se lei è un genitore, un membro della famiglia, o una persona che si prende cura della persona con autismo.

1. Qual è la sua posizione lavorativa? (per favore, segni tutte le voci pertinenti)

| Occupato (a pagamento, ivi compreso l'apprendistato / stage o altri tipi di formazione) | ⭘ |
| --- | --- |
| Occupato (non retribuito, compreso l'apprendistato / stage, altra formazione o attività di volontariato) | ⭘ |
| Categoria protetta | ⭘ |
| Disoccupato - ma disposto a lavorare | ⭘ |
| Disoccupato – e non disposto a lavorare | ⭘ |
| In pensione – e non coinvolto in attività lavorative | ⭘ |
| Casalinga/Casalingo- e non coinvolto in attività lavorative | ⭘ |
| Studente a tempo pieno - e non coinvolto in attività lavorative | ⭘ |
| Altro (specificare) | ⭘ |
|  |  |

1. Se impiegato (con retribuzione), quante ore a settimana lavora?

|  |
| --- |

1. Se impiegato (senza retribuzione), quante ore a settimana lavora?

|  |
| --- |

1. Se impiegato o studente a tempo pieno, ha fatto qualche assenza dal lavoro / luogo di studio nel corso degli ultimi 6 mesi perchè doveva prendersi cura della persona nello spettro autistico?

| No | ⭘ |
| --- | --- |
| Si | ⭘ |
| 35b. Se sì, specifichi per favore quante volte negli ultimi 6 mesi | |
|  | |

1. In media, quante ore a settimana impiega nel prendersi cura della persona nello spettro autistico?
2. 7. Ha lei (genitore/familiare/caregiver) usufruito di alcuni servizi sanitari o di assistenza sociale nel corso degli ultimi 6 mesi poichè si è preso cura della persona nello spettro autistico? Se sì, si prega di specificare quante volte ne ha usufruito nel corso degli ultimi 6 mesi, quale è stata la media dell’appuntamento/contatto e, se noto, il costo.

| Tipo di servizio | Numero di volte in cui ha utilizzato il servizio negli utimi 6 mesi | Durata (ore) | Pagamento  (Si/No) | Se si, indichi costo per visita (euro) |
| --- | --- | --- | --- | --- |
| Visita aggiuntiva con il medico di famiglia |  |  |  |  |
| Pianificazione familiare |  |  |  |  |
| Servizi sociali |  |  |  |  |
| Servizi psichiatrici |  |  |  |  |
| Consulente matrimoniale |  |  |  |  |
| Counselling |  |  |  |  |
| Gruppi di auto-aiuto |  |  |  |  |
| Consulenze |  |  |  |  |
| Altro (specificare la tipologia) | Numero di volte in cui ha utilizzato il servizio negli utimi 6 mesi | Durata (ore) | Pagamento  (Si/No) | Se si, indichi costo per visita (euro) |
|  |  |  |  |  |
|  |  |  |  |  |
|  |  |  |  |  |
|  |  |  |  |  |

Il questionario è finito. La ringraziamo per la sua partecipazione!
